# Succession Planning in the Iranian Health System: A Case Study of the Ministry of Health and Medical Education

**DOI:** 10.5539/gjhs.v6n5p174

**Published:** 2014-05-30

**Authors:** Mohamad Mehrtak, Soodabeh Vatankhah, Bahram Delgoshaei, Arian Gholipour

**Affiliations:** 1 Department of Health Services Management, School of Health Management and Information Sciences, Iran University of Medical Sciences, Tehran, Iran; 2 Public Administration Department, University of Tehran, School of Management, Tehran, Iran

**Keywords:** succession planning, health management, health system, public health, Iran

## Abstract

**Background:**

Succession planning promotes the culture of private ownership, staff loyalty to the organization and develops organizational commitment, and increases organizational stability. The study was conducted to examine the status of succession planning in the Iranian health system in order to highlight the key concepts, provide new insight, and attract the attention of senior managers of the Ministry of Health and Medical Education to the importance of succession planning in achieving organizational goals.

**Methods:**

In a qualitative study with a framework analysis approach, semi-structured interviews were conducted with a sample selected using purposive and snowball sampling procedure. The MAXQDA-10 was used to apply the codes and manage the data. The codes were extracted using inductive and deductive methods.

**Results:**

Fourteen themes and six main subthemes were identified, including planning, organizational culture, system approach, competency model, career path, and senior managers. Our findings indicate a lack of succession planning in the Iranian health system.

**Conclusion:**

lack of succession planning could lead to inefficiency and ineffectiveness in health services provision. Implementation of succession planning could maximize human resources utilization.

## 1. Introduction

Today, most organizations have a system in place to ensure leadership continuity in key positions, retain and develop intellectual and knowledge capital for the future, and encourage individual advancement (Rothwell, 2010). With the advent and aggressive expansion of the global marketplace, organizations are faced with increased pressure to identify individuals who possess the skills and qualifications necessary to lead the organizations in the challenging today’s environment. Many senior leaders have learned that the distinguishing characteristic of successful organizations is the ability to identify, develop, and deploy exceptional leadership talent (Barner, 2006).

Succession planning is the process of leading the organization by filling different management positions with capable internal and external people. The experiences of advanced countries show that succession planning is a time-consuming and costly process which requires systematic planning and efforts (Helton & Jackson, 2007).

Mamprin states that succession planning is the preparation to replace one leader with another. He argues that succession process is one of the greatest challenges an organization has to face (Abasi-Harafte, 2010).

Succession planning must be geared toward the strategic plans of an organization (Wiesman & Baker, 2013). It is recommended that organizations use a structured plan and proper evaluation tools for implementing the succession process (Krauss, 2008).

Succession planning process, organizational strategy, leadership, and culture are intertwined and provide an overview of corporate vision. Thus, an important factor in the long-term sustainability and viability of organizations is how well succession planning is integrated into their strategy, leadership development culture, and change management (Mandi, 2008).

Succession planning has become a strategic priority in health care organizations (Dye, 2005). Inexperience of student healthcare managers is an important barrier to succession planning in retiring senior and middle managers (Blouin, McDonagh et al., 2006).

One aim of succession planning is to match the organization’s available talent to its needed future talent. Another aim is to help the organization meet the strategic and operational challenges facing it by having the right people at the right place and at the right time to do the right things (Kim, 2006). In the new perspective of succession planning, each person in an organization is a leader (Beyers, 2006).

Rothwell emphasizes on the support and participation of senior managers in succession planning. He also reiterates that succession planning must involve all the levels of the organization (Rothwell, 2010).

Barner distinguishes succession planning from replacement planning and argues that the aim of succession planning is to identify, retain, and prepare managers who can be dependable replacements in emergency situations (Barner, 2006). In the succession planning spectrum, replacement is at one end and succession planning is at the other end. Replacement is the process of identifying appropriate alternatives for high-level management of the organization. This is simply a prediction of the future performance of individuals. In this approach, the assumption is that the present manager is an appropriate model for future managers, which does not seem to be true in today’s uncertain environment, characterized by fast-changing strategies and processes (Rothwell, 2010).

### 1.1 Iranian Context

To establish a merit system in the selection and appointment of people to professional management positions, Iran’s Civil Service Management Code requires executive agencies to determine the qualifications necessary to promote people to higher levels of management. High Council of Administration’s act on selection of managers defines our studied population as non-political and at the middle management level, and requires such qualifications as academic degree, personal morals as well as behavior, and group and organizational behavior (Supreme Council Office [SCO], 2011).

Managers are appointed for a four-year period and any transfer during this period occurs based on the results of performance appraisal, recommendations of officials, or with the approval of Administrative Reforms Commissions. If the selected individual is deemed incompetent during the first six months following appointment, the supervising manager can lay off the individual without any due process, but after this period it is necessary to follow the aforementioned process (SCO, 2003).

People must be selected based on their skill set and experience (Ley, 2002), but executive agencies are also required to incorporate ethical and behavioral qualifications into account in selection, appointment, and replacement of managers. Such qualifications include commitment to the Constitution, piety, courage, conscientiousness, intelligence and tolerance (SCO, 2011).

Executive agencies are obligated to establish and implement a performance management system that could evaluate the performance and productivity of the organization, the management, and the employees. It is also obligatory to provide a report of the performance of these agencies (Government of Islamic Republic of Iran [GIRI], 2007). It is thus necessary to create a succession database that can help officials and CEOs in identifying, developing, and retaining individuals with the potential to fill management positions (SCO, 2003; GIRI, 2007).

Executive agencies are also required to motivate employees by selecting internal people for management positions (SCO, 2003). These agencies can select 15% of managers from competent external pools of candidates who have expertise and experience pertinent to the job. The period of service in professional management positions is four years and can be extended (GIRI, 2007).

The purpose of this study was to highlight the key concepts, provide new insights, and increase the awareness of the senior managers of the Ministry of Health and Medical Education (MOHME) regarding the crucial importance of succession planning in achieving organizational goals.

## 2. Materials and Methods

The present qualitative study used the conceptual framework of succession planning developed by Mandi (2008). Semi-structured interviews were conducted based on a guide on succession planning. The interview guide was reviewed and revised using the views of the members of the research team.

The study population consisted of the general managers and heads of the departments of MOHME. The study population was comprised of 48 key managers of the Ministry. Twenty three key managers were selected using purposive and snowball sampling method. The first participant was selected based on his administrative position. Interviews were conducted in the office of each participant and lasted for 40 to 90 minutes. Informed consent was taken from the participants to record the interviews, which were immediately transcribed and typed by the researcher. Data were analyzed using MAXQDA-10 software based on conceptual framework analysis.

The validity and reliability of the research was examined based on four factors: credibility, dependability, confirmability, and transferability. Credibility was verified by ensuring that interviews were conducted and transcribed in a manner acceptable to the participant. Also the transcription of the interviews and extracted themes and subthemes were provided to experts in the field and a number of participants in order to provide their corrective comments.

Triangulation was used to achieve dependability (Boswell & Cannon, 2012). Accordingly, the data were collected by conducting interviews, recording them, and taking notes. Environmental triangulation was also used by selecting the sample from different key positions and different departments (Boswell & Cannon, 2012; Speziale, Streubert, & Carpenter, 2011).

As for confirmability, all the activities, including the procsses and the collected data were carefully recorded and the themes extracted during each interview were verified by the interviewee (Speziale, Streubert, & Carpenter, 2011). Finally, to ensure transferability, the research findings were reviewed and approved by six faculty members (Speziale, Streubert, & Carpenter, 2011).

As for ethical considerations, the approval of the Research Ethics Committee to conduct the study was obtained. During the interviews, the participants were assured that the data would be used for research purposes only and that their personal information would remain confidential. The interviewees were assured that they could withdraw from the study at any point.

## 3. Results

All the participants were male. They did not officially hold any managerial positions, but were temporarily assigned to these positions informally. Because the participants are key managers in MOHME, their demographic information is not provided in this research.

Six codes and 14 themes were identified (Table 1).

**Table 1:** Identified codes and themes

Themes	Subthemes
Planning	Current manager selection method
	Economic attitude
	Training program for candidates

Organizational culture	Political and ideological orientation
	Fairness in the selection process

Systems approach	Coordination between the succession planning system and other processes
	Condition of the comprehensive information system

Competency model	Criteria for candidate selection
	Physician-centered approach
	Competency indices

Career path	Identification of the current management potential of the candidates
	Performance appraisal

Senior managers	Guiding the employees to advance in their career paths
	Senior managers’ belief in succession planning

### 3.1 Planning

The majority of the participants confirmed the inconsistent performance and lack of planning in selection and appointment of people to key management positions. For example, one interviewee commented:

“Different departments use very different criteria. Sometimes the criterion is past experience in the field, sometimes it’s academic degree, and often it’s familiarity with the senior manager. There’s no clear method for identifying successors.”

Another interviewee argued:

“We don’t have such a thing as succession planning in the Ministry of Health. One senior manager may appoint me as their assistant just because of my looks and this continues at other levels of the organization.”

Resource management in an environment lacking succession planning was another issue highlighted by the participants. Duplication of effort, trial and error, and the subsequent waste of resources were evident in the interviews. Another interviewee stated:

“If there were succession planning, we would have a transfer of knowledge instead of waste of resources. We wouldn’t reinvent the wheel! You have often noted that a policy is established, then a person comes along and ignores it. After five years, another individual realizes that the previous policy was good and implements it again. There are so many instances of this nature. Well, this is a waste of resources. More importantly, human resources are the most expensive resources. We spend a lot of time and money on training people and we don’t use their services. This is the biggest loss we have ever experienced.”

### 3.2 Organizational Culture

Individuals’ opinions play an important role in their being selected as senior managers. Interviewees seem to criticize the favoritism in the appointment process. In this respect, one manager argued:

“In our country, appointing people to management positions hasn’t been based on their qualifications and professional capabilities. Instead, their social influences would be the determining factor in their appointments. Therefore the expertise and experience of candidates are less important criteria. This situation, however, is strongly reprimanded by the country’s leader and high ranking officials, who emphasize on equal opportunities for all.”

In fact, high ranking officials prefer to appoint people whom they trust regardless of their capabilities. Ethical commitments are far more important than professional expertise this is considered as a core value in the Iranian social system. An interviewee commented:

“Appointments are not practically based of the applicant’s knowledge and expertise; rather connections play an important role in this respect.”

### 3.3 Systems Approach

Lack of systems thinking in the departments of the Ministry has been considered as an important barrier to implementation of succession planning. The need for coordination between the succession planning system and other processes was emphasized by the participants. One interviewee stated:

“Some young men have been appointed as senior managers. They are great people, but they lack system thinking, which is a big problem in the Ministry. A certain individual comes to the Ministry and works there for a period of time. They focus on the present time and don’t care about what’s going to happen when they leave.”

The participants believed that a comprehensive information system on human resources in the MOHME is a must, which is a prerequisite to creating a stable managerial system and succession planning. One participant commended:

“Lack of a database for managers is a major problem in our health system. A database of competent managers could prevent management instability. It can also help institutionalize succession planning.”

### 3.4 Competency Model

Non-physician participants were critical of the influence of Physicians and the attention they received for high-level management positions. They considered this an important barrier to implementation of succession planning. One interviewee stated:

“One of the weaknesses of the health system is that it’s physician-centered, both in the Ministry and in universities. A number of chancellors of medical universities and policymakers believe that a good physician makes a good manager. But this is not true. Heath is a multidisciplinary and multi-factorial phenomenon. Studying medicine doesn’t empower people in all areas. We have a physician-centered view of our processes and systems. This attitude is a barrier to establishing a merit system.”

Another participant who was a physician looked at the issue from another perspective and believed that it is necessary to use public health physicians in key management positions. He said:

“I think it’s good to assign physicians who don’t have clinical inclinations to different management positions and whose main concern is public interest. Now someone may have a PhD in anatomy, but still could have a public health mentality. Because the Ministry of Health is concerned with public welfare, I believe it’s not inappropriate to use physicians in management positions.”

### 3.5 Career Path

The participants emphasized on promotion decisions based on previous performance, experience, training, and other capabilities. They also highlighted the benefits of performance appraisal in identifying potential internal candidates. One participant commented on the lack of succession planning in the Iranian health system:

“One reason why we don’t have succession planning in the Ministry of Health is the absence of a career path system. This means that I, as a new employee, don’t know how many years I have to work and what skills and capabilities I need to have to become a manager in the organization. Well, obviously this can lead to instability.”

Another participant commented on the importance of performance appraisal:

“One major weakness in our country’s administrative structure is that we don’t have a scientific and professional performance appraisal system for managers. What does that mean? It means that we never evaluate the performance of our managers quantitatively. We can’t determine how much a manager has helped the organization move toward its goals since they took their position.”

### 3.6 Senior Managers

Senior managers’ support for succession planning was an issue discussed by the majority of the participants, underlining the conservative attitude of senior managers and their concern about jeopardizing their current status and position. One participant stated:

“Another barrier to the implementation of succession planning is that many managers fear that succession planning threatens their own position in the long run. In the macro-management system, many try to keep potential candidates at lower levels, which could hinder the growth of succession planning.”

The concern about job security and the consequent lack of support by senior managers have cultural roots. One participant commented:

“The fact that managers are worried about succession planning has cultural roots. Our country has experienced much instability in the past, creating a conservative culture in our society. This kind of culture has, to a large extent, affected organizations as well.”

## 4. Discussion

Succession planning encourages individual advancement, increases organizational commitment among employees, and creates stability in the organization (Bolton & Roy, 2004; McConnell, 2006; Wolf, Bradle, & Greenhouse, 2006; Currie, 2010). Although there are regulations for selecting and appointing managers in our country, succession planning indicators such as its incorporation in the strategic planning of the organization have not been considered. These regulations are retrospective and cannot predict the future management needs of organizations.

Rollins argues that succession planning can eliminate the need to recruit managers from outside of the organization, which could reduce employment costs, decrease the length of training and orientation, and lead to minimal business disruption (Rollins & Gina, 2002). According to the current regulations, the Iranian public sector can use external pools of candidates only for 15% of management positions, and the candidates must have relevant expertise and experience. Our findings show the regulations are not adequately implemented and discriminatory practices in the selection and appointment of managers are exercised.

Clear and objective criteria must be used in selecting managers from among potential candidates (Friedman & Stewart, 1986), and all the candidates must enjoy equal opportunities in the succession planning process without any discrimination (Bolton & Roy, 2004). Iran’s contemporary laws highlight the importance of selecting managers based on technical and ethical criteria. However, our findings suggest that employers’ personal preferences play a significant role in the selection of managers.

According to systems thinking, change in one area of the system affects other areas as well. Perceiving the succession process through systems thinking paradigm requires the integration of the other subsystems within the organization. Succession planning is a process that includes strategic and financial planning, and preparation of successors. In other words, factors such as organizational strategy, culture, change management, and political and economic environment will all affect the succession process (Mandi, 2008). Therefore, succession planning requires a systematic effort within the organization to identify, develop, and retain potential leaders (Abasi-Harafte, 2010). Our current laws and regulations are related to replacement plans rather than succession planning and are not incorporated into the strategic planning of organizations. These issues, as well as the lack of a holistic systems thinking, have hindered the implementation of succession planning in the Iranian health system.

Succession planning must map the career path of employees, That is, the process must specify the skills and capabilities required for the occupation of higher positions in the organization. Career paths should account for the interests and wishes of candidates (Nasehifar, Dehghanpour, & Sanjari, 2011). The rules and regulations support the use of career paths, but our findings indicate that it has not received enough attention in practice.

It must be noted that high-performing physicians are not necessarily good leaders. Managers must know that outstanding clinical performance does not guarantee high leadership potential. Rather, they should look for other characteristics such as emotional intelligence (Goleman, Boyatzis, & MacKee, 2004). Physicians and faculty members are the primary candidates for management positions in Iran, and non-physician participants in the present research are considered as a major weakness of the country’s health system. In a study in the US pharmacy schools, many of the interviewees felt that succession planning makes it possible for faculty members to occupy certain positions on temporary basis (Van-Amburgh et al., 2010).

Career path is an important issue in the Iranian laws and was underlined by the participants in the present research. Metz argues that the succession planning process should be designed in such a way as to identify interested and competent candidates and appoint them to managerial positions through the career path (Metz, 1998). Promotion within an organization has been supported by other studies. Organizations that focus on promoting internal candidates are reported to have higher financial returns (Aitchison, 2004; S. K. Collins & K. S. Collins, 2007; Dean et al., 2002; Redeker, 2004). Various studies have shown that internal candidates selected and developed through the career paths are more likely to succeed and have higher efficiency than those employed from external pools (Blouin, McDonagh et al., 2006; Aitchison, 2004; S. K. Collins & K. S. Collins, 2007; Redeker, 2004; Bayo-Moriones & Ortín-Ánge, 2006). However, we found that the Iranian health system lacks a career path system for selecting managers.

Studies have shown that evaluation of internal employees can be used to identify and develop potential candidates for management positions (Blouin, McDonagh et al., 2006; S. K. Collins & K. S. Collins, 2007; Fruth, 2003; Kim, 2003). This process must show how much promotion decisions are based on past performance, experience, training, and capabilities of employees (Friedman & Stewart, 1986). In spite of the great emphasis placed on the importance of evaluation of employees, our findings suggest that there is a lack of a scientific and professional system for evaluating managers.

Succession planning is bound to fail without the support of senior managers (Wiesman & Baker, 2013; Nasehifar, Dehghanpour & Sanjari, 2011; Smilansky, 2007; Groves & Kevin, 2007). According to Rothwell, managers do not agree to the implementation of a plan unless they realize its benefits or see its short-term outcomes (Rothwell, 2010). Wackerle claims that senior executives may act as immovable obstacles on the way of potential successors, thus discouraging qualified candidates and damaging valuable talents (Abasi-Harafte, 2010). In our study, the participants argued that managers are concerned about their position being jeopardized by succession planning.

## 5. Conclusion

Our findings suggest a lack of succession planning in the Iranian health system and show that even the simple replacement plan is not well implemented. Beyers concluded from the interviews that succession planning is a complex process that needs to be customized by organizations. He noted that its application in healthcare is largely driven by staff turnover and the realization that commitment and loyalty are valuable assets in an organization (Beyers, 2006). A contextualized employee development plan can remove the barriers to the implementation of succession planning. Our study revealed that certain irrelevant factors, such as personal preferences of employers play a crucial role in selecting and appointing candidates.

If the current senior managers have gone through an extensive succession planning and promoted through a well-defined career path, they will no longer believe that their position is in jeopardy. The prevailing uncertainty in the promotion of individuals to higher management positions, which is due to a lack of a systematic performance appraisal system, has hindered the institutionalization of succession planning. We recommend that future research focus on identifying the barriers to implementation of succession planning.

## Appendix

***Interview Questions***

1. What is the current condition of succession planning in the organization? (Are there any similar plans?)
2. What are the advantages and disadvantages of succession planning?
3. What role do senior managers play in facilitating or hindering succession plans?
4. How important is the senior managers’ support of succession plans?
5. List the strengths, weaknesses, opportunities, and threats associated with succession planning.
6. How does the existing organizational culture affect the implementation of succession planning?
7. What competencies, skills, and characteristics do potential candidates need to possess?
8. How can potential successors be identified and evaluated?
9. How can political or ideological orientations affect succession planning?
10. What is your idea about the role of physicians and faculty members as potential candidates for key management positions in Ministry of Health and Medical Education?
